# Ocular Dominance is Associated With Interocular Asymmetry in Refractive Error and Axial Length Across Childhood and Early Adulthood

**DOI:** 10.1007/s44402-026-00155-8

**Published:** 2026-07-29

**Authors:** Síofra C. Harrington, Gareth Lingham, James Loughman, Ian Flitcroft, David A. Mackey, Michael Moore

**Affiliations:** 1 School of Physics, Clinical and Optometric Sciences, Technological University Dublin, Dublin, Ireland; 2 Centre for Eye Research Ireland (CERI), Sustainability and Health Research Hub (SHRH), Technological University Dublin, Dublin, Ireland; 3 Centre for Ophthalmology and Visual Sciences (incorporating Lions Eye Institute), University of Western Australia, Perth, Australia

**Keywords:** Anisometropia, Axial length, Interocular asymmetry, Myopia, Ocular dominance, Refractive error

## Abstract

**Purpose:**

To determine whether ocular dominance is associated with systematic interocular asymmetry in spherical equivalent refraction (SER) and axial length (AL) across childhood and early adulthood, and assess whether such asymmetry exceeds laterality effects.

**Methods:**

Data were analysed from three population-based cohorts: 728 children (6–7-year-olds) and 898 adolescents (12–13-year-olds) from Ireland and 303 young adults (25–30-year-olds) from Western Australia. Ocular dominance was assessed using sighting tests. SER was measured by cycloplegic autorefraction and AL by optical biometry. Interocular differences were evaluated using paired comparisons, concordance correlation coefficients (CCC), Deming regression, Bland–Altman analyses and mediation modelling. Analyses were stratified by refractive status and anisometropia.

**Results:**

Across all age groups, right–left differences in SER and AL were minimal (mean differences <0.05 D and <0.05 mm in children and adolescents), with small, significant laterality effects observed only in young adults (−0.13 D in SER; 0.06 mm in AL). In contrast, dominant–non-dominant comparisons demonstrated consistent directional asymmetry: the dominant eye was less hyperopic/more myopic (−0.09 D at 6–7 years; −0.10 D at 12–13 years; −0.08 D at 25–30 years) and longer (0.03–0.06 mm; all *p* < 0.05). Deming regression and Bland–Altman analyses confirmed systematic deviation from interocular symmetry, most pronounced in childhood. Mediation analyses indicated that ocular dominance statistically explained over 85% of the observed interocular asymmetry, independent of laterality. Dominance-related asymmetry was most evident in non-myopes (SER −0.09 to −0.36 D; AL 0.03–0.06 mm), attenuated in myopia and amplified in anisometropia (SER up to −1.09 D; AL up to 0.42 mm), particularly in younger cohorts.

**Conclusion:**

In this cross-sectional analysis, ocular dominance was associated with small but systematic interocular asymmetry in SER and AL across childhood and early adulthood, which exceeds laterality effects. Dominance-related asymmetry was attenuated in myopia and amplified in anisometropia. Ocular dominance should be considered when analysing interocular refractive and biometric data in research and clinical contexts.

Key Points

- Ocular dominance accounted for the majority (85%) of systematic interocular asymmetry in refractive error and axial length, exceeding simple right–left laterality effects.
- Ocular dominance was associated with subtle but systematic interocular differences, with the dominant eye being slightly longer and less hyperopic.
- Although small, these dominance-related differences approach the magnitude of axial length changes considered meaningful in myopia research, and may influence normative modelling and single-eye study designs.

## Introduction

Symmetry is typically considered a fundamental aspect of biological function; however, asymmetry is an inherent characteristic of many biological systems, including the human visual system [1]. Structural and functional differences between fellow eyes challenge the long-standing assumption of ocular symmetry that underpins much clinical practice and research [1]. Emerging evidence suggests that interocular asymmetries arise early in development and are associated with differences in ocular structure and function [1]. A central feature of this asymmetry is ocular dominance, a complex, multifactorial phenomenon driven by cortical processing that remains incompletely understood [2].

Previous research has demonstrated that interocular asymmetry and ocular dominance are associated with refractive error and ocular growth patterns [3–5], challenging the conventional practice of analysing or reporting data from a single eye in research studies [6]. This approach risks overlooking subtle yet clinically relevant differences that may influence visual outcomes and conditions such as myopia.

Recognising interocular asymmetry is important for establishing normative data and may be relevant when interpreting laterality-related findings in population-level trends, such as the right-eye predominance observed in retinal detachment studies [7]. Mahroo et al. [3], in a large population-based study of UK participants, reported that right eyes tended to be longer and more myopic than left eyes. Their analyses of more than 3800 individuals across four cohorts suggested that such asymmetries were not random but were influenced by ocular dominance. Importantly, they cautioned that reliance on right-eye data alone may introduce systematic bias.

However, findings from clinic-based studies have been less consistent. Jiang et al. [8] reported that the dominant eye tended to be longer and more myopic, particularly among anisometropic individuals, although participants without anisometropia were evaluated less extensively, whereas Cheng et al. [5] and Ito et al. [4] observed greater myopia and longer axial length (AL) in the non-dominant eye, particularly in anisometropic patients. The latter studies, though informative, were limited by clinic-based convenience samples of individuals with clinically significant myopia, raising concerns about generalisability.

Collectively, these findings highlight the need to characterise the relationship between ocular dominance and interocular asymmetry across different stages of visual development. Such evidence is essential for refining normative datasets and distinguishing typical developmental variation from clinically meaningful asymmetry.

The present study examines associations between ocular dominance, spherical equivalent refraction (SER) and AL in three cohorts: 728 schoolchildren (aged 6–7 years) and 898 adolescent schoolchildren (aged 12–13 years) in Ireland and 303 young adults (aged 25–30 years) in Western Australia. By analysing both eyes and stratifying by refractive status, this study provides insight into age-related patterns of interocular asymmetry across childhood, adolescence and early adulthood. While cross-sectional in design, the inclusion of a wide age range offers a developmental perspective on how dominance-related asymmetry in refractive and biometric measures varies from childhood into early adulthood.

## Methods

Data were drawn from participants in the Ireland Eye Study (IES) [9,10] and the Kidskin Young Adult Myopia Study (KYAMS). The IES included 1626 children recruited between June 2016 and January 2018, comprising 728 children aged 6–7 years (377 males, 351 females) and 898 adolescents aged 12–13 years (504 males, 394 females). The KYAMS sample comprised 303 young adults aged 25–30 years (116 males, 187 females), of whom 291 had complete paired refractive and AL data and were included in analyses. The participants were originally recruited as 5–6-year-old pupils from 33 primary schools in Perth, Western Australia (1995–1996) and subsequently re-contacted in adulthood between 2015 and 2018 using electoral rolls, telephone listings and social media [11].

A study flow diagram outlining participant recruitment, cohort stratification and inclusion in the interocular asymmetry analyses is provided in Supplementary Fig. S1.

In the IES, ocular dominance was assessed using the hole-in-the-card test (Dolman’s method) [12]. Participants held a 25 × 15 cm card containing a central 3 cm aperture at arm’s length and fixated the examiner’s nose through the opening. The fixing eye was observed by the examiner and recorded as the dominant eye. The procedure was repeated three times and the eye most consistently identified as dominant was designated as the dominant eye. In the KYAMS, ocular dominance was assessed using the hole-in-the-hands method, fixing on a 6/60 letter on a visual acuity chart at 4 m, repeated once. Hand dominance was recorded as the hand used for drawing in the IES and by self-report in KYAMS.

Refractive error was measured under cycloplegia in both studies. In the IES, cycloplegia was induced with one drop of topical anaesthetic (proxymetacaine hydrochloride 0.5%) followed by one drop of cyclopentolate hydrochloride 1% in participants with pale irises (blue or grey) and two drops of cyclopentolate 1% (administered 5 min apart) in participants with brown, green or hazel irises. Adequate cycloplegia was confirmed at least 20 minutes post-instillation by the absence of the pupillary light reflex and accommodative amplitude < 2.00 D. Autorefraction was performed using a Dong Yang Rekto ORK-11 autorefractor (Everview Corp.,everview.kr).

In KYAMS, cycloplegia was induced with one drop 1% tropicamide and autorefraction was performed 20 min later using a Nidek ARK-510A autorefractor (Nidek Co. Ltd., nidek.com). Cycloplegia was assessed using the pupillary light response and a second drop of 1% tropicamide was administered if necessary. SER was calculated as sphere plus half the cylinder. Myopia was defined as SER ≤ –0.50 D and hyperopia as SER ≥ +2.00 D on a per-eye basis, consistent with prior epidemiological studies [9, 13,14]. Anisometropia was defined as an interocular SER difference of ≥1.00 D [15].

AL was measured in both cohorts using the Zeiss IOLMaster 500 (Carl Zeiss Meditec AG, zeiss.com/meditec).

Five measurements were obtained per participant and the mean value was used for analysis.

### Statistical methodology

All statistical analyses were performed using IBM SPSS Statistics for Windows, Version 31 (ibm.com) and RStudio (Version 4.2.2; r-project.org). Data distribution was assessed using the Kolmogorov–Smirnov test and visual inspection of histograms. Continuous variables are presented as means and standard deviations (SD) and categorical variables as frequencies and percentages. A two-sided *p*-value < 0.05 was considered statistically significant.

#### Missing data

Complete-case analysis was performed; missing data were minimal (<4%).

To assess the robustness of findings, complementary analytical approaches were undertaken, including agreement analyses using Lin’s concordance correlation coefficients (CCC) and Bland–Altman methods, Deming regression to account for measurement error in both eyes and mediation modelling to disentangle dominance from laterality effects. These analyses functioned as sensitivity analyses to evaluate whether findings were consistent across different statistical frameworks.

Analyses focused on interocular asymmetry in SER and AL across three age cohorts (6–7, 12–13 and 25–30 years). Comparisons were conducted between right and left eyes and between dominant and non-dominant eyes.

##### Comparison of paired ocular data

Paired-sample *t*-tests were used for approximately normally distributed AL data, while the Wilcoxon signed-rank test was applied to non-normally distributed SER data.

##### Evaluation of interocular asymmetry

Mean interocular differences and agreement were assessed for right versus left eyes and for dominant versus non-dominant eyes. Agreement analyses considered both systematic error (bias) and random error (precision) [16–18].

Agreement between paired ocular measurements was assessed using:

- Pearson correlation coefficients to evaluate linear association,
- Lin’s CCC to quantify agreement by jointly assessing accuracy and precision [19] and
- Bland–Altman plots to visualise mean interocular differences (bias) and 95% limits of agreement (mean ± 1.96 SD).

Separate analyses were conducted for SER and AL within each age cohort.

##### Regression analyses

Deming regression was used to account for measurement error in both paired ocular measures, enabling assessment of systematic deviation and proportional bias. The models assumed equal measurement error in both eyes (error variance ratio *λ* = 1).

*Mediation analyses*: Statistical mediation analyses were conducted to quantify the extent to which ocular dominance explained observed interocular differences in SER and AL. Nonparametric mediation models with 1000 bootstrapped resamples were used to estimate the average causal mediation effect (ACME), the average direct effect (ADE), the total effect and the proportion of the total effect statistically mediated. Analyses were stratified by age cohort and performed separately for SER and AL.

The mediation framework was implemented using a two-stage generalised linear modelling structure that explicitly incorporated both right- and left-eye measures (e.g., SER_RE and SER_LE). Accordingly, estimated mediation effects of ocular dominance were derived while accounting for eye laterality within the model structure.

Given the cross-sectional design, mediation results were interpreted as explanatory rather than causal. The STROBE reporting guideline was used to draft this manuscript [20] and the STROBE reporting checklist [21] is included in Supplement A.

## Results

### Participant Characteristics and Dominance Distribution

Right-eye dominance and right-handedness were more common than left-eye dominance and left-handedness across all age cohorts (Table 1). Ocular dominance was not associated with sex in any cohort (all *χ*² tests, *p* > 0.44). In contrast, males were more likely than females to be left-handed in all age groups: 6–7 years (14.6% vs. 10.0%), 12–13 years (14.9% vs. 9.2%) and 25–30 years (15.5% vs. 7.0%; all *p* < 0.05).

**Table 1:** Demographic and refractive characteristics of participants (sex, handedness, ocular dominance, ethnicity and refractive status) across the three age cohorts.

	6–7 years	12–13 years	25–30 years
Sex— *n* (% male)	377 (51.8)	504 (56.1)	116 (38.3)
RE dominant—*n* (%)	496 (68.1)	587 (65.4)	194 (64.0)
R-handed—*n* (%)	635 (87.2)	787 (87.6)	272 (89.9)
Ethnicity—*n* (% white)	647 (88.9)	794 (88.4)	248 (81.8)
Myope (SER ≤ −0.50 D)—*n* (%)	26 (3.6)	204 (22.7)	99 (34.0)
Emmetrope (>−0.50 D, <+2.00 D)—*n* (%)	503 (69.1)	602 (67.0)	184 (63.2)
Hyperope (≥+2.00 D)—*n* (%)	199 (27.3)	92 (10.2)	8 (2.8)
Anisometropia (IOD ≥ 1D)—*n* (%)	53 (7.3)	92 (10.2)	21 (7.2)

*D* dioptre, *IOD* interocular difference, *n* number, *R* right, *RE* right eye, *SER* spherical equivalent refraction.

**Fig. 1:**
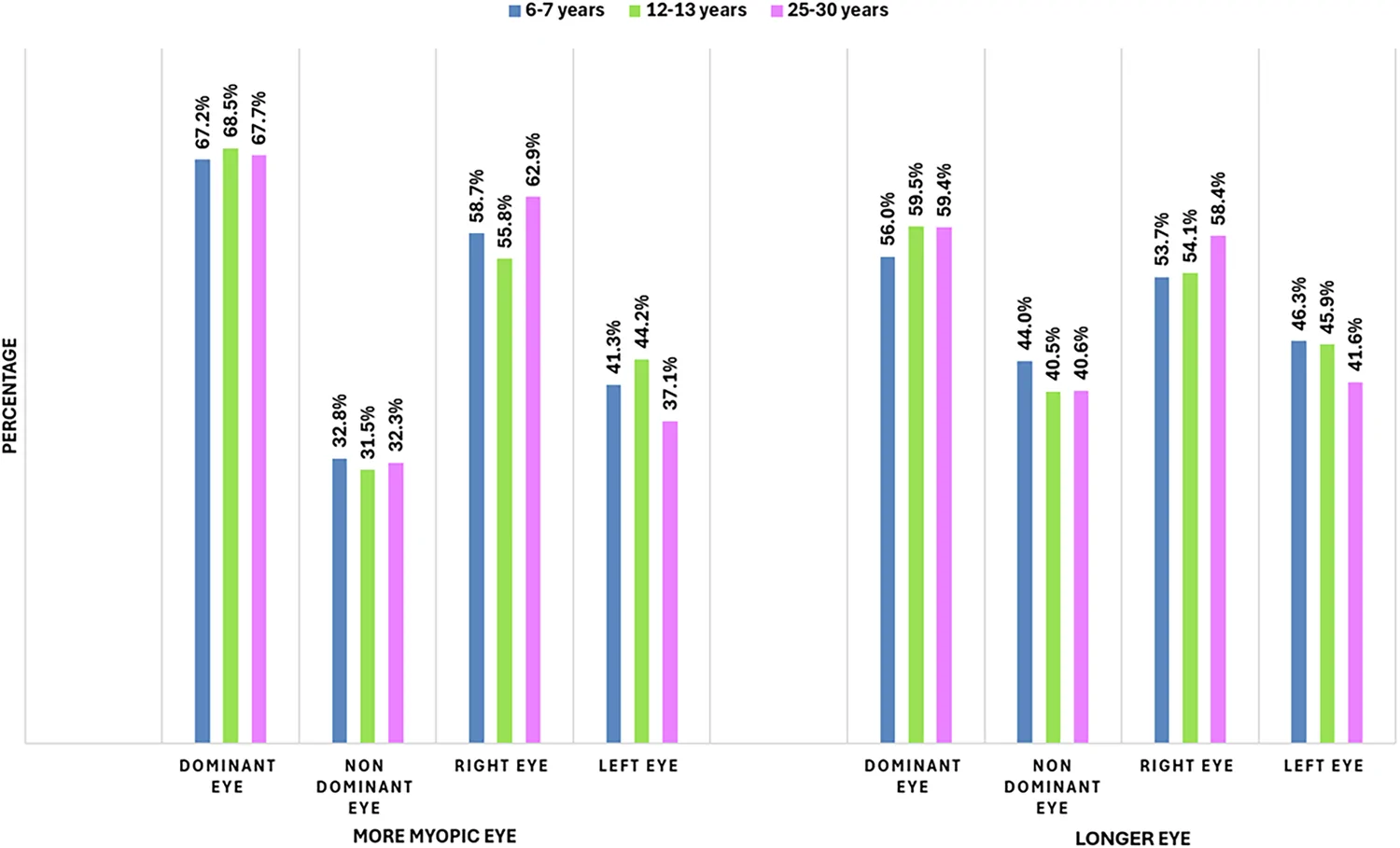
Distribution of the more myopic eye and the longer eye across age cohorts. Bar charts show the proportion of participants in whom the dominant eye or right eye was identified as the more myopic eye (right panel) and the longer eye (left panel) in three age groups: 6–7 years (*n* = 728), 12–13 years (*n* = 898) and 25–30 years (*n* = 291). Percentages are calculated within each age cohort. Departures from an expected 50:50 distribution were evaluated using *χ*² tests, demonstrating a higher-than-expected prevalence of the dominant eye as both the more myopic and longer eye across all cohorts, whereas right–left eye comparisons showed weaker directional asymmetry.

Categorical analyses demonstrated directional alignment of asymmetry in SER and AL with both ocular dominance and eye laterality, although the association was stronger for dominance. The dominant eye was longer in 56–59% of individuals and more myopic in ~67% across age cohorts. Right–left comparisons also showed a directional bias (~54–59%), with the right eye more frequently longer and/or more myopic, but with a smaller effect size than that observed for dominance. The distribution of the more myopic eye and the longer eye by ocular dominance and laterality across age cohorts is shown in Fig. 1.

Interocular differences in SER and AL (right–left and dominant–non-dominant) did not differ by sex (all *p* > 0.20). SER did not differ between males and females for any eye category (right, left, dominant or non-dominant; all *p* > 0.29). However, AL was significantly longer in males than females in all cohorts (6–7 years: mean difference 0.497 mm, *F* = 79.94, *p* < 0.001; 12–13 years: 0.506 mm, *F* = 77.50, *p* < 0.001; 25–30 years: 0.395 mm, *F* = 10.11, *p* = 0.002). Therefore, subsequent analyses combined sexes.

**Fig. 2:**
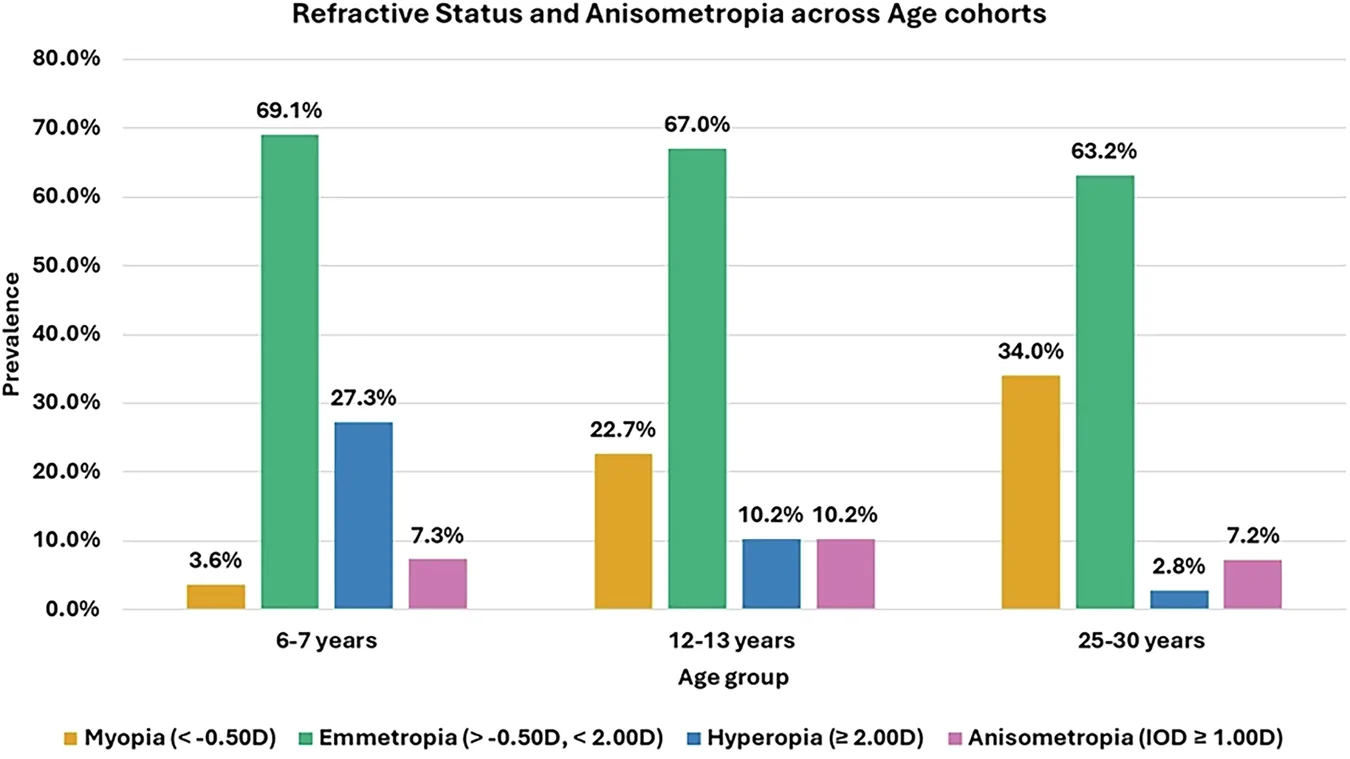
Prevalence of refractive status and anisometropia across age cohorts. Prevalence of myopia (spherical equivalent refraction [SER] ≤ −0.50 D), emmetropia (−0.50 D < SER < +2.00 D), hyperopia (SER ≥ +2.00 D) and anisometropia (interocular SER difference ≥ 1.00 D) in participants aged 6–7 years (*n* = 728), 12–13 years (*n* = 898) and 25–30 years (*n* = 291). Bars represent percentages within each age cohort.

Table 1 summarises demographic and refractive characteristics. Myopia prevalence increased with age, reaching 34% in young adults, while hyperopia prevalence was highest in the youngest cohort (27.3%). The prevalence of anisometropia was relatively stable across age groups, at approximately 7–10% (Fig. 2).

#### Interocular differences: right vs. left eyes

Mean SER differences between right and left eyes were small and not statistically significant in children (6–7 years: −0.019 D; 12–13 years: 0.026 D; both *p* > 0.48). In young adults, the right eye was significantly more myopic than the left eye, although the magnitude was small (−0.13 D, *p* = 0.002).

Mean AL showed strong right–left symmetry across all cohorts. In children and adolescents, mean interocular differences were ≤0.03 mm and not statistically significant. In young adults, a small but significant right–left asymmetry was observed (mean difference 0.06 mm; *p* < 0.05), with the right eye being longer on average.

Descriptive statistics for SER and AL in the right and left eyes are presented in Table 2.

**Table 2:** Descriptive statistics for spherical equivalent refraction (SER) and axial length (AL) in participants’ right, left, dominant and non-dominant eyes.

Age group		Mean	Std. deviation	Min	Max	*p*-value
6–7 years	SER RE (D)	1.391	1.252	−4.50	8.88	
	SER LE (D)	1.410	1.287	−5.00	8.88	
	SER DIFF RE–LE (D)	−0.019	0.638	−6.50	3.50	0.22
	SER DE (D)	1.398	1.203	−5.00	9.00	
	SER NDE (D)	1.487	1.325	−4.50	9.00	
	SER DIFF DE–NDE (D)	−0.089	0.639	−6.50	2.25	**<0.001**
	AL RE (mm)	22.528	0.787	19.14	25.73	
	AL LE (mm)	22.525	0.824	19.50	27.01	
	Diff AL RE–LE (mm)	0.003	0.401	−4.34	2.42	0.41
	AL DE (mm)	22.543	0.779	19.69	25.73	
	AL NDE (mm)	22.510	0.831	19.14	27.01	
	AL Diff DE–NDE (mm)	0.033	0.400	−3.96	4.34	**0.01**
12–13 years	SER RE (D)	0.340	1.620	−10.25	8.25	
	SER LE (D)	0.314	1.717	−7.88	8.75	
	SER DIFF RE–LE (D)	0.026	0.825	−5.50	6.00	0.17
	SER DE (D)	0.319	1.542	−10.25	8.25	
	SER NDE (D)	0.420	1.771	−7.75	8.75	
	SER DIFF DE–NDE (D)	−0.101	0.808	−5.50	6.00	**<0.001**
	AL RE (mm)	23.501	0.890	20.37	27.65	
	AL LE (mm)	23.487	0.916	20.07	27.48	
	Diff AL RE–LE (mm)	0.014	0.317	−3.05	1.87	0.10
	AL DE (mm)	23.519	0.883	20.37	27.65	
	AL NDE (mm)	23.469	0.922	20.07	27.48	
	AL Diff DE–NDE (mm)	0.053	0.314	−3.05	2.00	**<0.001**
25–30	SER RE (D)	−0.439	2.037	−11.50	8.00	
	SER LE (D)	−0.306	1.911	−10.50	7.875	
	SER DIFF RE–LE (D)	−0.133	0.733	−8.63	1.75	**0.001**
	SER DE (D)	−0.411	1.991	−11.50	7.88	
	SER NDE (D)	−0.335	1.960	−10.50	9.00	
	SER DIFF DE–NDE (D)	−0.077	0.741	−8.63	2.38	**0.04**
	AL RE (mm)	23.641	1.067	19.40	28.02	
	AL LE (mm)	23.582	1.048	19.55	27.47	
	Diff AL RE_LE (mm)	0.059	0.262	−0.69	3.08	**<0.001**
	AL DE (mm)	23.631	1.052	19.55	27.92	
	AL NDE (mm)	23.592	1.063	19.40	28.02	
	AL Diff DE–NDE (mm)	0.039	0.266	−0.69	3.08	**0.006**

Participants were 728 aged 6–7 years, 898 aged 12–13 years and 303 aged 25–30 years. Significant relationships are highlighted in boldface.

*AL* axial length, *DIFF* difference, *D* dioptre, DE dominant eye, *LE* left eye, *mm* millimetres, *NDE* non-dominant eye, RE right eye, *SER* spherical equivalent refraction, *Std.* standard.

### Interocular differences: dominant vs. non-dominant eyes

Across all age groups, the dominant eye was, on average, slightly less hyperopic/more myopic than the non-dominant eye (6–7 years: −0.09 D, *p* = 0.004; 12–13 years: −0.101 D, *p* < 0.001; 25–30 years: −0.077 D, *p* = 0.04). Similarly, AL was significantly longer in the dominant eye in all cohorts: 0.033 mm at 6–7 years (*p* = 0.01), 0.053 mm at 12–13 years (*p* < 0.001) and 0.039 mm at 25–30 years (*p* = 0.01). Descriptive statistics for SER and AL in dominant and non-dominant eyes are presented in Table 2.

#### Agreement and regression analyses

Agreement between paired eyes was high across all cohorts (Table 3). Lin’s CCC increased with age for both SER and AL (SER CCC: 0.87 at 6–7 years to 0.97 in adults; AL CCC: 0.88–0.97), indicating increasing interocular symmetry with age.

**Table 3:** Relationship between spherical equivalent refraction (SER) and axial length (AL) in right vs. left eyes and dominant vs. non-dominant eyes among 728 participants aged 6–7 years, 898 participants aged 12–13 years and 303 participants aged 25–30 years.

Age-group	Lin’s CCC coefficient	Pearson coefficient	*R*²	Deming regression equation
*SER, right eye (X) vs. left eye (Y)*				
6–7 years	0.874	0.874	0.764	*Y* = −0.03 + 1.03 * *X*
12–13 years	0.879	0.879	0.773	*Y* = -0.05 + 1.07 * *X*
25–30 years	0.929	0.966	0.933	*Y* = 0.10 + 0.93* *X*
*SER, dominant eye (X) vs. non-dominant eye (Y)*				
6–7 years	0.875	0.881	0.776	*Y* = −0.07 + 1.12 * *X*
12–13 years	0.888	0.889	0.790	*Y* = 0.05 + 1.18 * *X*
25–30 years	0.967	0.964	0.930	*Y* = 0.07 + 0.98 * *X*
*AL right eye (X) vs. left eye (Y)*				
6–7 years	0.876	0.877	0.769	*Y* = −1.21 + 1.05 * *X*
12–13 years	0.938	0.939	0.882	*Y* = −0.76 + 1.03 * *X*
25–30 years	0.939	0.984	0.969	*Y* = 0.40 + 0.98 * *X*
*AL dominant eye (X) vs. non-dominant eye (Y)*				
6–7 years	0.877	0.878	0.771	*Y* = −1.74 + 1.08 * *X*
12–13 years	0.938	0.940	0.884	*Y* = −1.15 + 1.05 * *X*
25–30 years	0.967	0.984	0.968	*Y* = −0.33 + 1.01 * *X*

*CCC* concordance correlation coefficient, *X* dominant eye, *Y* non-dominant eye.

Agreement was marginally lower for dominant–non-dominant eye comparisons than for right–left eye comparisons, particularly in childhood. Deming regression analyses demonstrated slopes close to unity and intercepts near zero for right–left comparisons across all cohorts, consistent with strong symmetry (Supplementary Fig. S1).

For dominant–non-dominant comparisons, greater deviation from the line of identity was observed in younger cohorts. At 6–7 years of age, regression slopes exceeded 1.1 and intercepts deviated further from zero (SER −0.07 D; AL −1.74 mm), indicating proportional asymmetry favouring the dominant eye. Intercept values in Deming models reflect scaling parameters within the regression framework and should not be interpreted as literal interocular biometric offsets. These deviations were smaller in adolescence and approached unity (slope) and zero (intercept) in young adulthood.

#### Interocular agreement and regression analyses

Table 3 summarises the relationship between SER and AL for both right and left eyes, as well as dominant and non-dominant eyes. The table includes Lin’s CCC, Pearson correlation coefficients, *R*² values and Deming regression equations, which collectively quantify the agreement, strength of correlation and proportionality of these relationships.

Although dominance-related mean differences were small and within expected measurement variability, they were consistently larger than laterality-related differences and demonstrated a consistent directional pattern across age groups.

#### Bland–Altman analyses

**Fig. 3:**
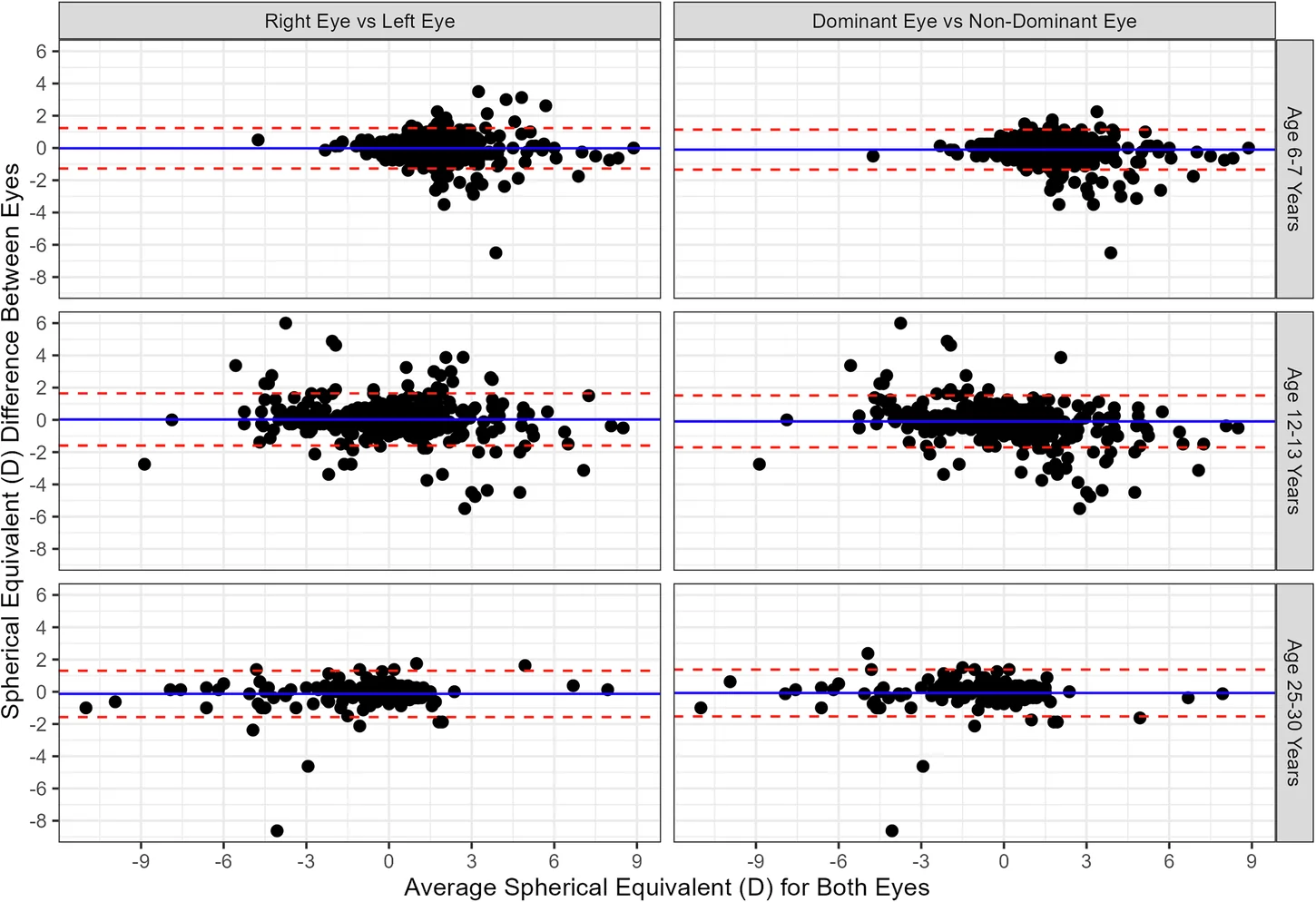
Bland–Altman plots for spherical equivalent refraction (SER). Bland–Altman plots showing interocular differences in SER for right versus left eyes (left panels) and dominant versus non-dominant eyes (right panels) in participants aged 6–7 years (top row), 12–13 years (middle row) and 25–30 years (bottom row). Solid lines indicate mean interocular differences (bias) and dashed lines represent the 95% limits of agreement (LOA; mean ± 1.96 SD). The mean SER difference (upper and lower LOAs) between the right eye vs. the left eye was −0.018 (1.237, −1.272) D for 6–7-year-olds, −0.026 (1.644, −1.592) D for 12–13-year-olds and −0.133 (1.556, −1.622) D for 25–30-year-olds. The corresponding mean differences (95% CIs) between the dominant and non-dominant eyes were −0.100 (1.142, −1.340), −0.096 (1.512, −1.705) and −0.077 (1.337, −1.742) D, respectively.

**Fig. 4:**
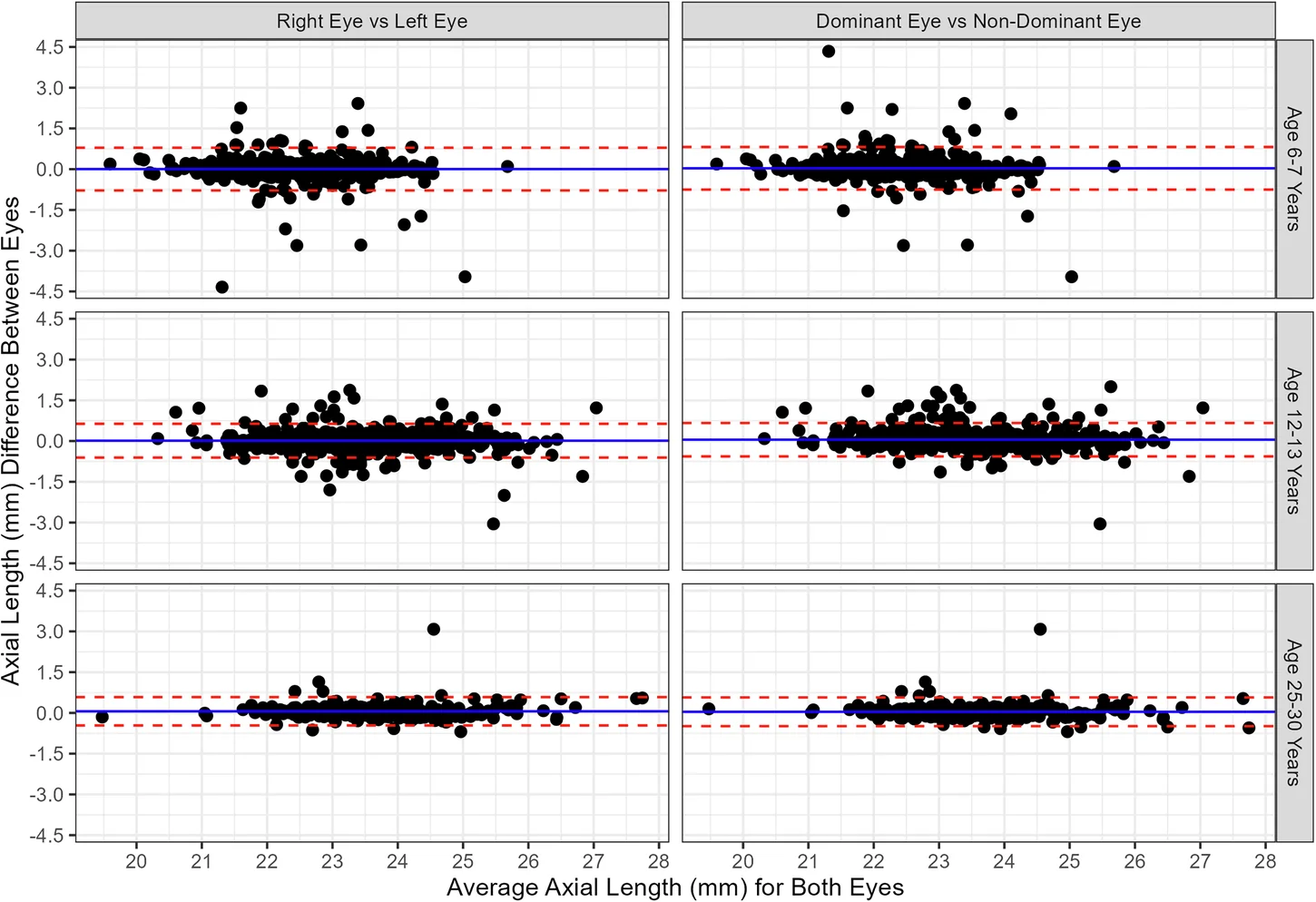
Bland–Altman plots for axial length (AL). Bland–Altman plots showing interocular differences in AL for right versus left eyes (left panels) and dominant versus non-dominant eyes (right panels) in participants aged 6–7 years (top row), 12–13 years (middle row) and 25–30 years (bottom row). Solid lines indicate mean interocular differences (bias) and dashed lines represent the 95% limits of agreement (mean ± 1.96 SD). The solid blue lines represent the mean differences and the dotted red lines represent the upper and lower 95% limits of agreement. The mean AL difference (95% confidence intervals (CI)) between the right eye vs the left eye was 0.0033 (0.790, −0.783) mm for 6–7-year-olds, 0.014 (0.636, −0.609) mm for 12–13-year-olds and 0.059 (0.756, −0.694) mm for 25–30-year-olds. The corresponding mean difference (95% CIs) between the dominant and non-dominant eyes was 0.033 (0.817, −0.751), 0.050 (0.665, −0.655) and 0.039 (0.780, −0.655) mm, respectively.

Bland–Altman plots supported the regression findings (Figs. 3, 4). For right–left comparisons, mean differences in SER and AL were close to zero, and limits of agreement were narrow across all cohorts, indicating strong interocular symmetry.

For dominant–non-dominant comparisons, mean differences were consistently larger than those observed for right–left comparisons, supporting the presence of a small but systematic dominance-related bias. Mean SER differences ranged from **−**0.09 to **−**0.20 D and AL differences from 0.03 to 0.07 mm. Although these differences were small and generally within expected measurement variability, dominance-related asymmetry showed a consistent directional pattern across cohorts.

#### Mediation analyses

Mediation models quantified the extent to which ocular dominance statistically explained observed interocular differences in SER and AL. The two-stage generalised linear modelling framework incorporated both right- and left-eye measures (SER_RE and SER_LE), such that effects attributed to dominance were estimated while accounting for eye laterality.

For SER, the ACME of ocular dominance was 0.91 (*p* < 0.001) at 6–7 years, 0.94 (*p* < 0.001) at 12–13 years and 0.78 (*p* < 0.001) in young adults. Direct effects were small or non-significant. Across cohorts, more than 88% of the total interocular difference in SER was statistically mediated by dominance, independent of laterality.

For AL, ACME values were similarly high (6–7 years: 0.93; 12–13 years: 0.98; 25–30 years: 0.89; all *p* < 0.001), with negligible direct effects. In all age groups, over 93% of the total interocular difference was statistically mediated by dominance, again independent of laterality. Given the cross-sectional design, these findings reflect statistical decomposition rather than causal inference.

#### Stratification by refractive status

To examine whether dominance-related asymmetry varied by refractive profile, analyses were stratified by refractive status. When stratified by refractive status (Tables 4, 5), dominance-related asymmetry was most consistently observed in non-myopic participants and was particularly evident among anisometropic individuals across all age groups.

**Table 4:** Differences in axial length (AL) between right vs. left eyes and dominant vs. non-dominant eyes, stratified by refractive status.

Age group (years)	Refractive status	Axial length difference	Mean difference	Std. dev.	Std. error	*t*	*p*-value
6–7	Myopes (≤−0.50 D)	AL RE–AL LE	0.044	0.179	0.035	1.248	0.11
		AL DE–AL NDE	0.033	0.182	0.036	0.929	0.18
	Non-myopes (>−0.50 D)	AL RE–AL LE	0.002	0.407	0.015	0.114	0.46
		AL DE–AL NDE	0.033	0.406	0.015	2.150	**0.02**
	Emmetropes (>−0.50, <2.00 D)	AL RE–AL LE	−0.018	0.383	0.017	−1.077	0.14
		AL DE–AL NDE	−0.009	0.384	0.017	−0.500	0.31
	Hyperopes (≥+2.00 D)	AL RE–AL LE	0.053	0.460	0.033	1.618	0.05
		AL DE–AL NDE	0.139	0.441	0.031	4.407	**<0.001**
	Anisometropes (IOD ≥ 1D)	AL RE–AL LE	−0.056	1.122	0.154	-0.364	0.36
		AL DE–AL NDE	0.415	1.042	0.143	3.005	**0.002**
	Non-anisometropes (IOD < 1D)	AL RE–AL LE	0.008	0.275	0.011	0.750	0.23
		AL DE AL NDE	0.003	0.275	0.011	0.266	0.40
12–13	Myopia (≤−0.50 D)	AL RE–AL LE	−0.002	0.459	0.032	−0.075	0.47
		AL DE–AL NDE	0.017	0.459	0.032	0.535	0.30
	Non-myopes (>−0.50 D)	AL RE–AL LE	0.018	0.262	0.010	1.835	**0.03**
		AL DE–AL NDE	0.059	0.256	0.010	6.094	**<0.001**
	Emmetropes (>−0.50, <+2.00 D)	AL RE–AL LE	0.012	0.179	0.007	1.578	0.06
		AL DE–AL NDE	0.022	0.178	0.007	3.062	**0.001**
	Hyperopes (≥+2.00 D)	AL RE–AL LE	0.062	0.555	0.058	1.076	0.14
		AL DE–AL NDE	0.301	0.470	0.049	6.143	**<0.001**
	Anisometropes (IOD ≥ 1 D)	AL RE–AL LE	−0.041	0.808	0.084	-0.490	0.31
		AL DE–AL NDE	0.248	0.770	0.080	3.088	**0.001**
	Non-anisometropes (IOD < 1 D)	AL RE–AL LE	0.020	0.195	0.007	2.882	**0.002**
		AL DE–AL NDE	0.027	0.194	0.007	3.945	**<0.001**
25–30	Myopia (≤−0.50 D)	AL RE–AL LE	0.095	0.392	0.039	2.415	**0.009**
		AL DE–AL NDE	0.056	0.399	0.040	1.395	0.08
	Non-myopes (>−0.50 D)	AL RE–AL LE	0.042	0.166	0.012	3.477	**<0.001**
		AL DE–AL NDE	0.033	0.168	0.012	2.723	0.004
	Emmetropes (>−0.50, <2.00 D)	AL RE–AL LE	0.037	0.131	0.010	3.831	**<0.001**
		AL DE–AL NDE	0.019	0.135	0.010	1.902	**0.03**
	Hyperopes (≥+2.00 D)	AL RE–AL LE	0.145	0.535	0.189	0.767	0.23
		AL DE–AL NDE	0.355	0.407	0.144	2.466	**0.02**
	Anisometropes (IOD ≥ 1 D)	AL RE–AL LE	0.382	0.783	0.171	2.238	**0.02**
		AL DE–AL NDE	0.3471	0.800	0.175	1.989	**0.03**
	Non-anisometropes (IOD < 1 D)	AL RE–AL LE	0.035	0.148	0.009	3.854	**<0.001**
		AL DE–AL NDE	0.017	0.151	0.009	1.845	**0.03**

Paired samples *t*-tests were used to assess statistical significance, with significant differences observed in dominance-related asymmetry for non-myopes in younger cohorts (*p* < 0.05). Significant associations highlighted in boldface.

*AL* axial length, *DIFF* difference, *D* Dioptre, *DE* dominant eye, *LE* left eye, *mm* millimetres, *NDE* non-dominant eye, *RE* right eye, *Std*. standard.

**Table 5:** Differences in spherical equivalent refraction (SER) between right vs. left eyes and dominant vs. non-dominant eyes across three age cohorts (6–7, 12–13 and 25–30 years), stratified by refractive status.

Age group (years)	Refractive status	SER difference	Mean difference (dioptres)	Std. dev.	Std. error	*Z*	*p*-value
6–7	Myopes (≤-0.50 D)	SER RE–SER LE	0.048	0.310	0.061	−0.981	0.33
		SER DE–SER NDE	−0.087	0.273	0.054	−1.634	0.11
	Non-myopes (>-0.50 D)	SER RE–SER LE	−0.021	0.647	0.024	−0.349	0.73
		SER DE – SER NDE	−0.089	0.648	0.024	−2.662	**<0.001**
	Emmetropes (>−0.50, <2.00 D)	SER RE– SER LE	0.004	0.334	0.015	0.276	0.39
		SER DE–SER NDE	−0.021	0.335	0.015	−1.404	0.08
	Hyperopes (≥+2.00 D)	SER RE–SER LE	−0.084	1.092	0.077	−1.086	0.14
		SER DE–SER NDE	−0.300	1.053	0.075	−4.025	**<0.001**
	Anisometropes (IOD ≥ 1D)	SER RE–SER LE	−0.241	2.022	0.278	−0.866	0.20
		SER DE–SER NDE	−1.092	1.714	0.235	−4.638	**<0.001**
	No anisometropes (IOD < 1 D)	SER RE–SER LE	−0.001	0.346	0.013	−0.101	0.46
		SER DE–SER NDE	−0.021	0.345	0.013	−1.557	0.06
12–13	Myopes (≤−0.50 D)	SER RE–SER LE	0.077	1.119	0.078	0.984	0.16
		SER DE–SER NDE	0.106	1.117	0.078	1.349	0.09
	Non-myopes (>−0.50 D)	SER RE – SER LE	0.011	0.717	0.027	−1.221	0.22
		SER DE–SER NDE	−0.151	0.702	0.027	−4.963	**<0.001**
	Emmetropes (>-0.50, <2.00 D)	SER RE–SER LE	0.032	0.364	0.015	2.169	0.**02**
		SER DE–SER NDE	−0.038	0.365	0.015	−2.528	**0.006**
	Hyperopes (≥+2.00 D)	SER RE–SER LE	−0.129	1.736	0.181	−0.713	0.24
		SER DE–SER NDE	−0.925	1.472	0.153	−6.030	**<0.001**
	Anisometropes (IOD ≥ 1 D)	SER RE–SER LE	0.143	2.359	0.246	0.580	0.28
		SER DE–SER NDE	−0.707	2.190	0.228	−3.098	**0.001**
	Non-anisometropes (IOD < 1 D)	SER RE– SER LE	0.013	0.358	0.013	0.994	0.16
		SER DE–SER NDE	−0.031	0.357	0.013	−2.498	**0.006**
25–30	Myopes (≤−0.50 D)	SER RE–SER LE	−0.217	1.158	0.116	−1.867	0.**03**
		SER DE–SER NDE	−0.091	1.175	0.118	−0.770	0.22
	Non-myopes (>−0.50 D)	SER RE–SER LE	−0.090	0.349	0.025	−4.648	**<0.001**
		SER DE–SER NDE	−0.069	0.354	0.026	−2.006	**0.004**
	Emmetropes (>−0.50, <2.00 D)	SER RE–SER LE	−0.077	0.265	0.020	−3.936	**<0.001**
		SER DE–SER NDE	−0.041	0.272	0.020	−2.064	0.**02**
	Hyperopes (≥+2.00 D)	SER RE– SER LE	−0.391	1.181	0.418	−0.936	0.19
		SER DE–SER NDE	−0.703	1.002	0.354	−1.985	0.**04**
	Anisometropes (IOD ≥ 1 D)	SER RE–SER LE	−0.964	2.420	0.528	−1.826	**0.04**
		SER DE – SER NDE	−0.821	2.475	0.540	−1.521	0.07
	Non-anisometropes (IOD < 1 D)	SER RE–SER LE	-0.069	0.292	0.0178	−3.852	**<0.001**
		SER DE–SER NDE	−0.0185	0.300	0.0182	−1.015	0.16

Statistical significance was assessed using the Wilcoxon signed-rank test, with significant differences highlighted for dominance-related asymmetry in non-myopes across all age groups (*p* < 0.05). Statistically significant associations highlighted in boldface.

*DIFF* difference, *D* Dioptre, *DE* dominant eye, *LE* left eye, *mm* millimetres, *NDE* non-dominant eye, *RE* right eye, *SER* spherical equivalent refraction, *Std*. standard.

In non-myopes, dominant eyes were significantly less hyperopic/more myopic and longer across all age groups (SER differences −0.07 to −0.16 D; AL differences 0.03–0.06 mm in children; all *p* < 0.02) suggesting that dominance-related asymmetry may be most evident before the onset of established myopia. In myopes, dominance-related asymmetry in SER and AL was inconsistent or absent across cohorts. Among hyperopes, the dominant eye was more myopic and longer in several age groups (e.g., SER −0.30 D at 6–7 years, *p* < 0.001; AL + 0.30 mm at 12–13 years, *p* < 0.001). Across all refractive groups, right–left differences were minimal, with small laterality effects emerging only in young adults. Detailed estimates for each refractive subgroup are provided in Tables 4, 5.

The distribution of the longer eye and the more myopic eye by ocular dominance and laterality across refractive categories is shown in Table 6.

**Table 6:** Distribution of the longer eye and the more myopic eye by ocular dominance and laterality across age cohorts and refractive categories.

Age category	Refractive category	DE is longer than NDE	RE is longer than LE	DE is more myopic than NDE	RE is more myopic than LE
6–7 years					
	Myope	16 (61.5%)	17 (65.4%)	21 (80.8%)	13 (50.0%)
	Non-myope	388 (55.7%)	374 (53.3%)	468 (66.7%)	414 (59.0%)
	Emmetrope	269 (53.9%)	264 (52.5%)	339 (67.4%)	297 (59.0%)
	Hyperope	119 (60.4%)	110 (55.3%)	129 (64.8%)	117 (58.8%)
	Anisometropia	48 (72.7%)**	39 (59.1%)	46 (69.7%)	32 (48.5%)
	Non-anisometropia	356 (54.3%)	352 (53.2%)	443 (66.9%)	395 (59.7%)
12–13 years					
	Myope	109 (53.4%)	103 (50.5%)	122 (59.8)%	117 (57.4%)
	Non-myope	425 (61.2%)*	383 (55.2%)	493 (71.0%)**	384 (55.3%)
	Emmetrope	356 (59.1%)	329 (54.7%)	418 (69.4%)	333 (55.3%)
	Hyperope	**69 (75.0%) (0.002)**	54 (58.7%)	75(81.5%)***	51 (55.4%)
	Anisometropia	59 (63.4%)	44 (47.3%)	62 (66.7%)	32 (48.5%)
	Non-anisometropia	475 (59.0%)	442 (54.9%)	553 (68.7%)	395 (59.7%)*
25–30 years					
	Myope	58 (58.6%)	60 (60.6%)	53 (53.5%)	55 (55.6%)
	Non-myope	114 (59.4%)	114 (59.4%)	130 (67.7%)**	142 (74.0%)***
	Emmetrope	106 (57.6%)	110 (59.8%)	123 (66.8%)	137 (74.5%)
	Hyperope	8 (100.0%)**	4 (50.0%)	7 (87.5%)*	5 (62.5%)**
	Anisometropia	14 (66.7%)	15 (71.4%)	13 (61.9%)	14 (66.7%)
	Non-anisometropia	158 (58.5%)	159 (58.9%)	170 (63.0%)	183 (67.8%)

Values are shown as the number (percentage) of participants in whom the dominant eye (DE) or right eye (RE) was the longer eye (axial length) or the more myopic eye (spherical equivalent refraction, SER) within each age cohort (6–7, 12–13 and 25–30 years) and refractive category. Percentages are calculated within each subgroup. Departures from an expected 50:50 distribution were assessed using *χ*² tests; significant *p*-values are shown in parentheses. Refractive categories were defined as myopia (SER ≤ −0.50 D), emmetropia (−0.50 D < SER < +2.00 D), hyperopia (SER ≥ +2.00 D), anisometropia (interocular SER difference ≥ 1.00 D) and non-anisometropia (interocular SER difference < 1.00 D). Significant differences are highlighted in boldface with *p < 0.05, **p < 0.01, ***p < 0.001.

*DE* dominant eye, *LE* left eye, *NDE* non-dominant eye, *RE* right eye.

## Discussion

This study examined the relationship between ocular dominance and interocular asymmetry in SER and AL across childhood (6–7 years), adolescence (12–13 years) and early adulthood (25–30 years). Across all cohorts, right and left eyes demonstrated strong symmetry in ocular parameters, with only small laterality-related differences emerging in adulthood. In contrast, dominant–non-dominant eye comparisons revealed more consistent asymmetries, with effects evident across refractive groups and larger effect sizes observed in non-myopic and anisometropic participants. Together, these findings indicate that ocular dominance is more strongly associated with systematic interocular asymmetry than eye laterality, particularly in younger age groups. The observed deviation from an expected 50:50 distribution, with the dominant eye more frequently identified as both the more myopic and the longer eye, indicates that interocular asymmetry is directionally aligned with ocular dominance rather than occurring at random. This directional bias complements the small but consistent mean differences observed and supports the interpretation that dominance-related asymmetry reflects structured biological variation rather than measurement noise.

### Ocular Dominance and Refractive Patterning

In non-myopic children and adolescents, the dominant eye was, on average, less hyperopic and closer to emmetropia than the non-dominant eye. This pattern is consistent with previous population-based studies reporting small but systematic dominance-related differences in refraction and AL [3]. Although the magnitude of these differences was modest, their consistency across age groups and analytical approaches suggests that dominance-related asymmetry represents a reproducible feature of typical refractive development rather than random variation.

These findings align with conceptual models proposing binocularly coordinated eye growth, in which interocular differences are regulated rather than eliminated [22,23]. Experimental evidence showing that the dominant eye preferentially guides fixation [24], accommodation [25] and vergence responses [26] provides a plausible functional context for such asymmetry. However, the present cross-sectional data do not establish a mechanistic role for dominance in emmetropisation, and the observed associations should be interpreted as developmental patterning rather than evidence of causal control.

### Modulation of Dominance-related Asymmetry in Myopia and Anisometropia

Dominance-related asymmetry was attenuated in myopic participants, whereas larger interocular differences were observed in anisometropic individuals. Across refractive categories and age cohorts, the dominant eye was more frequently identified as both the longer and the more myopic eye than expected by chance, whereas right–left eye comparisons showed weaker and less consistent deviations from a 50:50 distribution. In myopia, the dominant eye was not consistently closer to emmetropia, suggesting that progressive axial elongation may override the subtle dominance-related patterning evident during non-myopic refractive development. This attenuation is consistent with the idea that once myopia is established, binocular growth becomes increasingly driven by shared environmental and optical factors rather than by early dominance-related biases [22,23].

In contrast, anisometropic participants exhibited larger dominance-aligned interocular differences in both SER and AL, particularly in childhood and adolescence. While a difference in SER is expected by definition in anisometropia, the present findings show that this difference is directionally aligned with ocular dominance rather than occurring randomly between eyes. These findings indicate that anisometropia is associated with an amplification of dominance-related asymmetry rather than its absence. While previous studies have reported variable associations between dominance and refractive asymmetry [3–5], the present data suggest that ocular dominance continues to bias interocular refractive and biometric differences in anisometropia, albeit with greater variability.

Overall, dominance-related asymmetry was most clearly expressed in non-myopic refractive states, became less consistently detectable in myopia and was amplified in anisometropia rather than disrupted. Therefore, anisometropia appears to reflect exaggerated dominance-aligned eye growth rather than random or laterality-driven asymmetry. These findings are consistent with earlier reports showing impaired emmetropisation and increasing interocular asymmetry with age and myopia progression [24,25]. Although some studies have reported greater myopia in the non-dominant eye in specific clinical populations [4,5], the present results indicate that dominance-related asymmetry appeared more pronounced in anisometropic individuals.

### Comparison with Population-based Studies

The present findings closely mirror those reported in large population cohorts. Mahroo et al. [3] reported dominance-related differences of ~0.07 D in SER and 0.04–0.06 mm in AL in Avon Longitudinal Study of Parents and Children (ALSPAC) and TwinsUK participants; values comparable to the 0.09–0.20 D and 0.03–0.07 mm differences observed here. The consistency of effect sizes across independent cohorts, age ranges and study designs strengthens confidence that dominance-related asymmetry is a generalisable phenomenon rather than a cohort-specific artefact.

While Mahroo et al. [3] demonstrated dominance-aligned asymmetry in predominantly adult populations, the present study extends those observations in several important respects. First, by examining large population-based samples across childhood, adolescence and early adulthood, it provides a developmental perspective on how dominance-related asymmetry manifests across refractive maturation. Second, stratified analyses show that dominance effects are strongest in non-myopic eyes, attenuated in myopia and amplified in anisometropia, indicating modulation by refractive phenotype. Third, formal modelling disentangling dominance from laterality confirms that dominance accounts for the majority of systematic interocular asymmetry. Together, these additions clarify that dominance-related asymmetry represents a structured feature of refractive development rather than an isolated adult finding.

### Statistical Considerations and Interocular Asymmetry

Agreement, regression and mediation analyses demonstrated greater symmetry between the right and left eyes than between the dominant and non-dominant eyes. Although absolute differences in SER and AL were small and generally within measurement repeatability, the consistency of the direction of the effect across independent cohorts suggests that ocular dominance may contribute to structured interocular asymmetry rather than random variation.

Mediation analyses indicated that ocular dominance accounted statistically for a substantial proportion of the observed interocular differences in SER and AL, with indirect effects exceeding direct effects across all cohorts. As expected in a cross-sectional design, these findings reflect statistical decomposition rather than causal mediation. While categorical analyses demonstrated a modest right–left directional bias, the mediation framework explicitly incorporated laterality within the modelling structure. Importantly, the proportion of interocular difference statistically mediated by dominance remained high after accounting for laterality, indicating that dominance-related asymmetry cannot be explained solely by population-level right-eye predominance.

Although multiple subgroup analyses were performed without formal correction for multiplicity, the principal inferences are supported by sensitivity analyses demonstrating convergence of directional effects across paired comparisons, agreement analyses, Deming regression modelling and mediation frameworks. Key conclusions were based on consistent directional effects across cohorts rather than isolated subgroup p-values. Taken together, these complementary approaches support the interpretation that dominance-related asymmetry represents a structured, non-random component of interocular variation during refractive development. Although the differences were small and generally within expected measurement variability, the consistency of the direction of the effect across independent cohorts suggests that ocular dominance contributes to structured interocular asymmetry rather than random variation. However, the magnitude of the observed differences was modest and should not be interpreted as evidence of clinical significance at the individual level.

### Laterality Effects

Eye dominance and laterality were correlated, with the right eye being typically the dominant eye. Despite this, laterality-related differences in SER and AL were detected only in young adults and were small in magnitude. These findings suggest that laterality-related asymmetry may emerge with age, potentially reflecting cumulative environmental influences or developmental drift. However, the limited size and late emergence of these effects indicate that laterality plays a comparatively minor role in interocular asymmetry relative to ocular dominance.

### Methodological Considerations and Limitations

Strengths of this study include large, well-characterised cohorts spanning a wide age range and the use of multiple complementary analytical approaches. Limitations include reliance on a binary-sighting-dominance test, differences in geographic location between cohorts and the cross-sectional design.

The study relied on a sighting-dominance test, which yields a binary classification and may not capture dynamic or task-dependent aspects of sensory dominance. Recent work by Pepose et al. [27] using a binocular, head-mounted visual simulator, revealed substantial discordance between sighting and sensory dominance and highlighted that ocular ‘preference’ is dynamic, task-dependent and better expressed as dominance strength rather than laterality alone. Nevertheless, the present findings show that even this simple dominance measure captures measurable biometric asymmetries. Future studies incorporating quantitative or sensory-based dominance measures may provide deeper insight into the strength of dominance and its relationship to refractive development.

Additional limitations include geographic differences between cohorts, a smaller adult sample size and cohort-specific measurement protocols. The adult cohort may have been underpowered to detect small dominance effects within refractive subgroups. However, refractive error was measured under cycloplegia in all participants and the primary analyses focused on within-individual interocular differences, minimising the impact of between-cohort methodological variation. Finally, the cross-sectional design precludes inference about temporal or causal relationships.

### Implications for Research and Clinical Practice

Although dominance-related interocular differences were small and not clinically actionable at the individual level, their consistency across cohorts indicates a structured biological pattern rather than random variation. These findings demonstrate that interocular symmetry cannot be assumed in refractive research and reliance on single-eye analyses, particularly right-eye analyses, may introduce subtle but systematic bias into normative refractive and AL datasets, especially in paediatric populations. In clinical contexts, ocular dominance may reflect not only behavioural preference but also a modest structural bias; in individuals with long-standing dominance-aligned asymmetry; therefore, altering the refractive balance of the dominant eye during monovision correction may represent a larger functional shift than assumed, with potential implications for binocular balance and adaptation. Furthermore, in myopia management trials where AL changes of 0.03–0.05 mm are considered meaningful, dominance-related asymmetry may approach the magnitude of treatment effects and should be accounted for in study design and statistical modelling. The amplification of dominance-aligned asymmetry in anisometropia further suggests that binocular refractive development is directionally patterned rather than random. Thus, explicit consideration of ocular dominance in bilateral analyses and interventional studies may improve precision in refractive research and enhance clinical interpretation.

## Conclusion

Ocular dominance was more strongly associated with interocular asymmetry in refractive error and AL than eye laterality, particularly during childhood and adolescence. These dominance-aligned differences were small, attenuated in myopia and amplified in anisometropia, indicating that interocular refractive development is directionally patterned rather than random. Although not indicative of causal control, these findings identify ocular dominance as a consistent source of structured interocular variation that should be considered when analysing bilateral ocular data, constructing normative biometric models and interpreting small changes in AL in myopia research.

## Supplementary information

Supplementary Figures

STROBE checklist

## Data Availability

The data that support the findings of this study are not publicly available due to participant confidentiality and consent restrictions. De-identified data may be made available from the corresponding author upon reasonable request and with appropriate institutional and ethical approvals.
