# Supplementary material for: Ocular Dominance is Associated With Interocular Asymmetry in Refractive Error and Axial Length Across Childhood and Early Adulthood
Source: Ophthalmic Physiol Opt. 2026 Jul 29;46(5):1095–106. doi: 10.1007/s44402-026-00155-8 (PMC13545045; doi:10.1007/s44402-026-00155-8)
Supplement: Supplementary file 1 [file 44402_2026_155_MOESM1_ESM.docx]

**Supplementary Information: Ocular dominance is associated with interocular asymmetry in refractive error and axial length across childhood and early adulthood**

**Supplementary Figures**

**Figure S1: Study flow diagram.
Participant flow across three cohorts: 6–7-year-olds (n = 728) and 12–13-year-olds (n = 898) from the Ireland Eye Study (IES), and 25–30-year-olds from the Kidskin Young Adult Myopia Study (KYAMS; n = 303 examined, n = 291 included with complete paired data). Inclusion required ocular dominance classification and bilateral cycloplegic refractive and axial length measurements.**

Figure S2: Scatter plots and Deming regression for interocular comparisons

Scatter plots illustrating the relationship between paired ocular measurements for spherical equivalent refraction (SER). Comparisons are shown for right versus left eyes and dominant versus non-dominant eyes across the three age cohorts (6–7 years, 12–13 years, and 25–30 years). Solid lines represent Deming regression fits, and dashed lines indicate the line of identity.

Figure S3: Scatter plots and Deming regression for interocular comparisons

Scatter plots illustrating the relationship between paired ocular measurements for axial length (AL). Comparisons are shown for right versus left eyes and dominant versus non-dominant eyes across the three age cohorts (6–7 years, 12–13 years, and 25–30 years). Solid lines represent Deming regression fits, and dashed lines indicate the line of identity.
